# Supplementary material for: Potential demand for National Health Insurance in Zimbabwe: Evidence from selected urban informal sector clusters in Harare
Source: PLoS One. 2023 May 30;18(5):e0286374. doi: 10.1371/journal.pone.0286374 (PMC10228813; doi:10.1371/journal.pone.0286374)
Supplement: S1 File — (DOC) [file pone.0286374.s001.doc]

**QUESTIONNAIRE A: INFORMAL SECTOR PARTICIPANTS**

Ethics clearance reference number: 2019_DE_18(PD)_T CHIPUNZA

Research permission reference number:

Date_____________________ Location_____________________

Title: The Financing of Health Care in Zimbabwe: In Search of a Socially Inclusive Mechanism

**Dear Participant**

Thank you for participating in this study. Your contribution is highly appreciated. The response nearest what you think on the question will be chosen.

**A. Demographic & socioeconomic information**

1. . Gender

1. Male 0. Female

101. Age *______(completed years)*

102. Marital status

1. Married 0. Single/ Divorced/ Widowed

103. What is the highest level of education that you have completed?

1. No education 2. Primary 3. Secondary 4. Tertiary

104. How many members are in your household?____________*(number).*

105. Informal sector activity

1. Clothing & shoe sales 5. Metal business

2. Hardware store 6. Timber & furniture business

3. Food kiosk 7. Agricultural products

4. Repairs

Other *(Specify)*_______________________________

106. Period involved in informal sector*_______(years).*

107. You are involved in the informal sector as:

1. Own account worker 3. Employer

2. Employee 4. Contributing family member

108. How many people work in this business?________*(number)*

109. What is your average monthly-household-net-income from informal sector activities? (Zw$, **i.e. BONDS**)

1. <500 2. 500 to < 1000 3. 1000 to < 3000 4. ≥ 3000

**B. Healthcare-related information**

200. What is the distance to the nearest health facility in the area you live? *__________(km).*

201. How much did your household spend on health care during the last 3 months? (Zw$, **i.e. BONDS**)

1. 0 1 2.<100 3. 100 to < 500 4. 500 to < 1000 5. ≥ 1000

202. Has your household faced any difficulties paying for health care in the last 12 months?

1. Yes 0. No

203. What would you say about the cost of health care in Zimbabwe?

1. Affordable 2. Expensive 3. Very expensive

204. What would you say about the quality of public health care delivery in Zimbabwe?

4.Very good 3.Good 2. Satisfactory 1.Poor

**C. Health insurance-related information**

300. Do you know about health insurance/ medical aid societies in Zimbabwe?

1. Yes 0. No

301. Are you currently a member of a medical aid society?

1. Yes 0. No

302. Are you currently a member of a resource-pooling scheme (e.g. savings & lending club, burial society)?

1. Yes 0. No

303. Would you say that health insurance/ medical aid protects patients from high medical costs?

1. Yes 0. No 2. Not sure

304. Would you support the use of your money to pay health care expenses for someone who is sicker than yourself?

1. Yes 0. No

**D. Willingness to participate and pay for National Health Insurance**

Suppose government introduces a subsidized health insurance scheme for the informal sector whereby those who join and contribute some specified monthly premiums will not pay for hospitalisation and specialist treatment at public and private health facilities, consultation at medical practitioners, maternity, blood transfusion, dental care, laboratory tests, x-ray and scan services, and prescription drugs up to a family limit of Zw$40000 (**i.e. BONDS)** per year.

1. Would you be willing to participate in the scheme?

1.Yes [go to question (ii)]

0. No [go to section E ]

1. Would you be willing to pay Zw$50 (**i.e. BONDS)** per person per month?

Yes [go to question (iii)]

No [go to question (iv)]

1. Would you be willing to pay Zw$75 (**i.e. BONDS)** per person per month?

Yes [go to question (v)]

No [go to question (v)]

1. Would you be willing to pay Zw$25 (**i.e. BONDS)** per person per month?

Yes [go to section E ]

No [go to question (v)]

1. What is the maximum amount that you would be willing to pay per person per month? Zw$ (**i.e. BONDS)** ___________

**E. Suitable health care financing mechanism for Zimbabwe**

In your view, which is the best way to finance health care in Zimbabwe?

Joint responsibility between citizens & government (NHI) 1.

Direct (cash) payments when one needs health care (OOPP) 2. .

Private health insurance (medical aid societies) 3.

General government tax revenue 4.

**END OF QUESTIONNAIRE**

**QUESTIONNAIRE B: INFORMAL SECTOR PARTICIPANTS**

Ethics clearance reference number: 2019_DE_18(PD)_T CHIPUNZA

Research permission reference number:

Date_____________________ Location_____________________

Title: The Financing of Health Care in Zimbabwe: In Search of a Socially Inclusive Mechanism

**Dear Participant**

Thank you for participating in this study. Your contribution is highly appreciated. The response nearest what you think on the question will be chosen.

**A. Demographic & socioeconomic information**

1. . Gender

1. Male 0. Female

101. Age *______(completed years)*

102. Marital status

1. Married 0. Single/ Divorced/ Widowed

103. What is the highest level of education that you have completed?

1. No education 2. Primary 3. Secondary 4. Tertiary

104. How many members are in your household?____________*(number).*

105. Informal sector activity

1. Clothing & shoe sales 5. Metal business

2. Hardware store 6. Timber & furniture business

3. Food kiosk 7. Agricultural products

4. Repairs

Other *(Specify)*_______________________________

106. Period involved in informal sector*_______(years).*

107. You are involved in the informal sector as:

1. Own account worker 3. Employer

2. Employee 4. Contributing family member

108. How many people work in this business?________*(number)*

109. What is your average monthly-household-net-income from informal sector activities? (Zw$, **i.e. BONDS**)

1. <500 2. 500 to < 1000 3. 1000 to < 3000 4. ≥ 3000

**B. Healthcare-related information**

200. What is the distance to the nearest health facility in the area you live? *__________(km).*

201. How much did your household spend on health care during the last 3 months? (Zw$, **i.e. BONDS**)

1. 0 1 2.<100 3. 100 to < 500 4. 500 to < 1000 5. ≥ 1000

202. Has your household faced any difficulties paying for health care in the last 12 months?

1. Yes 0. No

203. What would you say about the cost of health care in Zimbabwe?

1. Affordable 2. Expensive 3. Very expensive

204. What would you say about the quality of public health care delivery in Zimbabwe?

4.Very good 3.Good 2. Satisfactory 1.Poor

**C. Health insurance-related information**

300. Do you know about health insurance/ medical aid societies in Zimbabwe?

1. Yes 0. No

301. Are you currently a member of a medical aid society?

1. Yes 0. No

302. Are you currently a member of a resource-pooling scheme (e.g. savings & lending club, burial society)?

1. Yes 0. No

303. Would you say that health insurance/ medical aid protects patients from high medical costs?

1. Yes 0. No 2. Not sure

304. Would you support the use of your money to pay health care expenses for someone who is sicker than yourself?

1. Yes 0. No

**D. Willingness to participate and pay for National Health Insurance**

Suppose government introduces a subsidized health insurance scheme for the informal sector whereby those who join and contribute some specified monthly premiums will not pay for hospitalisation and specialist treatment at public and private health facilities, consultation at medical practitioners, maternity, blood transfusion, dental care, laboratory tests, x-ray and scan services, and prescription drugs up to a family limit of Zw$40000 (**i.e. BONDS)** per year.

1. Would you be willing to participate in the scheme?

1.Yes [go to question (ii)]

0. No [go to section E ]

1. Would you be willing to pay Zw$75 (**i.e. BONDS)** per person per month?

Yes [go to question (iii)]

No [go to question (iv)]

1. Would you be willing to pay Zw$100 (**i.e. BONDS)** per person per month?

Yes [go to question (v)]

No [go to question (v)]

1. Would you be willing to pay Zw$50 (**i.e. BONDS)** per person per month?

Yes [go to section E ]

No [go to question (v)]

1. What is the maximum amount that you would be willing to pay per person per month? Zw$ (**i.e. BONDS)** ___________

**E. Suitable health care financing mechanism for Zimbabwe**

In your view, which is the best way to finance health care in Zimbabwe?

Joint responsibility between citizens & government (NHI) 1.

Direct (cash) payments when one needs health care (OOPP) 2. .

Private health insurance (medical aid societies) 3.

General government tax revenue 4.

**END OF QUESTIONNAIRE**

**QUESTIONNAIRE C: INFORMAL SECTOR PARTICIPANTS**

Ethics clearance reference number: 2019_DE_18(PD)_T CHIPUNZA

Research permission reference number:

Date_____________________ Location_____________________

Title: The Financing of Health Care in Zimbabwe: In Search of a Socially Inclusive Mechanism

**Dear Participant**

Thank you for participating in this study. Your contribution is highly appreciated. The response nearest what you think on the question will be chosen.

**A. Demographic & socioeconomic information**

1. . Gender

1. Male 0. Female

101. Age *______(completed years)*

102. Marital status

1. Married 0. Single/ Divorced/ Widowed

103. What is the highest level of education that you have completed?

1. No education 2. Primary 3. Secondary 4. Tertiary

104. How many members are in your household?____________*(number).*

105. Informal sector activity

1. Clothing & shoe sales 5. Metal business

2. Hardware store 6. Timber & furniture business

3. Food kiosk 7. Agricultural products

4. Repairs

Other *(Specify)*_______________________________

106. Period involved in informal sector*_______(years).*

107. You are involved in the informal sector as:

1. Own account worker 2. Employer

2. Employee 4. Contributing family member

108. How many people work in this business?________*(number)*

109. What is your average monthly-household-net-income from informal sector activities? (Zw$, **i.e. BONDS**)

1. <500 2. 500 to < 1000 3. 1000 to < 3000 4. ≥ 3000

**B. Healthcare-related information**

200. What is the distance to the nearest health facility in the area you live? *__________(km).*

201. How much did your household spend on health care during the last 3 months? (Zw$, **i.e. BONDS**)

1. 0 1 2.<100 3. 100 to < 500 4. 500 to < 1000 5. ≥ 1000

202. Has your household faced any difficulties paying for health care in the last 12 months?

1. Yes 0. No

203. What would you say about the cost of health care in Zimbabwe?

1. Affordable 2. Expensive 3. Very expensive

204. What would you say about the quality of public health care delivery in Zimbabwe?

4.Very good 3.Good 2. Satisfactory 1.Poor

**C. Health insurance-related information**

300. Do you know about health insurance/ medical aid societies in Zimbabwe?

1. Yes 0. No

301. Are you currently a member of a medical aid society?

1. Yes 0. No

302. Are you currently a member of a resource-pooling scheme (e.g. savings & lending club, burial society)?

1. Yes 0. No

303. Would you say that health insurance/ medical aid protects patients from high medical costs?

1. Yes 0. No 2. Not sure

304. Would you support the use of your money to pay health care expenses for someone who is sicker than yourself?

1. Yes 0. No

**D. Willingness to participate and pay for National Health Insurance**

Suppose government introduces a subsidized health insurance scheme for the informal sector whereby those who join and contribute some specified monthly premiums will not pay for hospitalisation and specialist treatment at public and private health facilities, consultation at medical practitioners, maternity, blood transfusion, dental care, laboratory tests, x-ray and scan services, and prescription drugs up to a family limit of Zw$40000 (**i.e. BONDS)** per year.

1. Would you be willing to participate in the scheme?

1.Yes [go to question (ii)]

0. No [go to section E ]

1. Would you be willing to pay Zw$100 (**i.e. BONDS)** per person per month?

Yes [go to question (iii)]

No [go to question (iv)]

1. Would you be willing to pay Zw$125 (**i.e. BONDS)** per person per month?

Yes [go to question (v)]

No [go to question (v)]

1. Would you be willing to pay Zw$75 (**i.e. BONDS)** per person per month?

Yes [go to section E ]

No [go to question (v)]

1. What is the maximum amount that you would be willing to pay per person per month? Zw$ (**i.e. BONDS)** ___________

**E. Suitable health care financing mechanism for Zimbabwe**

In your view, which is the best way to finance health care in Zimbabwe?

Joint responsibility between citizens & government (NHI) 1.

Direct (cash) payments when one needs health care (OOPP) 2. .

Private health insurance (medical aid societies) 3.

General government tax revenue 4.

**END OF QUESTIONNAIRE**

**QUESTIONNAIRE D: INFORMAL SECTOR PARTICIPANTS**

Ethics clearance reference number: 2019_DE_18(PD)_T CHIPUNZA

Research permission reference number:

Date_____________________ Location_____________________

Title: The Financing of Health Care in Zimbabwe: In Search of a Socially Inclusive Mechanism

**Dear Participant**

Thank you for participating in this study. Your contribution is highly appreciated. The response nearest what you think on the question will be chosen.

**A. Demographic & socioeconomic information**

1. . Gender

1. Male 0. Female

101. Age *______(completed years)*

102. Marital status

1. Married 0. Single/ Divorced/ Widowed

103. What is the highest level of education that you have completed?

1. No education 2. Primary 3. Secondary 4. Tertiary

104. How many members are in your household?____________*(number).*

105. Informal sector activity

1. Clothing & shoe sales 5. Metal business

2. Hardware store 6. Timber & furniture business

3. Food kiosk 7. Agricultural products

4. Repairs

Other *(Specify)*_______________________________

106. Period involved in informal sector*_______(years).*

107. You are involved in the informal sector as:

1. Own account worker 3. Employer

2. Employee 4. Contributing family member

108. How many people work in this business?________*(number)*

109. What is your average monthly-household-net-income from informal sector activities? (Zw$, **i.e. BONDS**)

1. <500 2. 500 to < 1000 3. 1000 to < 3000 4. ≥ 3000

**B. Healthcare-related information**

200. What is the distance to the nearest health facility in the area you live? *__________(km).*

201. How much did your household spend on health care during the last 3 months? (Zw$, **i.e. BONDS**)

1. 0 1 2.<100 3. 100 to < 500 4. 500 to < 1000 5. ≥ 1000

202. Has your household faced any difficulties paying for health care in the last 12 months?

1. Yes 0. No

203. What would you say about the cost of health care in Zimbabwe?

1. Affordable 2. Expensive 3. Very expensive

204. What would you say about the quality of public health care delivery in Zimbabwe?

4.Very good 3.Good 2. Satisfactory 1.Poor

**C. Health insurance-related information**

300. Do you know about health insurance/ medical aid societies in Zimbabwe?

1. Yes 0. No

301. Are you currently a member of a medical aid society?

1. Yes 0. No

302. Are you currently a member of a resource-pooling scheme (e.g. savings & lending club, burial society)?

1. Yes 0. No

303. Would you say that health insurance/ medical aid protects patients from high medical costs?

1. Yes 0. No 2. Not sure

304. Would you support the use of your money to pay health care expenses for someone who is sicker than yourself?

1. Yes 0. No

**D. Willingness to participate and pay for National Health Insurance**

Suppose government introduces a subsidized health insurance scheme for the informal sector whereby those who join and contribute some specified monthly premiums will not pay for hospitalisation and specialist treatment at public and private health facilities, consultation at medical practitioners, maternity, blood transfusion, dental care, laboratory tests, x-ray and scan services, and prescription drugs up to a family limit of Zw$40000 (**i.e. BONDS)** per year.

1. Would you be willing to participate in the scheme?

1.Yes [go to question (ii)]

0. No [go to section E ]

1. Would you be willing to pay Zw$25 (**i.e. BONDS)** per person per month?

Yes [go to question (iii)]

No [go to question (iv)]

1. Would you be willing to pay Zw$50 (**i.e. BONDS)** per person per month?

Yes [go to question (v)]

No [go to question (v)]

1. Would you be willing to pay Zw$10 (**i.e. BONDS)** per person per month?

Yes [go to section E ]

No [go to question (v)]

1. What is the maximum amount that you would be willing to pay per person per month? Zw$ (**i.e. BONDS)** ___________

**E. Suitable health care financing mechanism for Zimbabwe**

In your view, which is the best way to finance health care in Zimbabwe?

Joint responsibility between citizens & government (NHI) 1.

Direct (cash) payments when one needs health care (OOPP) 2. .

Private health insurance (medical aid societies) 3.

General government tax revenue 4.

**END OF QUESTIONNAIRE**
