# Supplementary material for: Potential demand for National Health Insurance in Zimbabwe: Evidence from selected urban informal sector clusters in Harare
Source: PLoS One. 2023 May 30;18(5):e0286374. doi: 10.1371/journal.pone.0286374 (PMC10228813; doi:10.1371/journal.pone.0286374)
Supplement: S2 File — (DOC) [file pone.0286374.s002.doc]

**CONSENT TO PARTICIPATE IN THIS STUDY**

I, _____________________________________________ (participant name), confirm that the person asking my consent to take part in this research has told me about the nature, procedure, potential benefits and anticipated inconvenience of participation.

I have read (or had explained to me) and understood the study as explained in the information sheet.

I have had sufficient opportunity to ask questions and am prepared to participate in the study.

I understand that my participation is voluntary and that I am free to withdraw at any time without penalty (if applicable).

I am aware that the findings of this study will be processed into a research report, journal publications and/or conference proceedings, but that my participation will be kept confidential unless otherwise specified.

I agree to the recording of the interview.

I have received a signed copy of the informed consent agreement.

Participant Name & Surname………………………………………… (please print)

Participant Signature……………………………………………..Date…………………

Researcher’s Name & Surname…CHIPUNZA TAMISAI…………………(please print)

Researcher’s signature……………………..…………………...Date…………………
