# Supplementary material for: Potential demand for National Health Insurance in Zimbabwe: Evidence from selected urban informal sector clusters in Harare
Source: PLoS One. 2023 May 30;18(5):e0286374. doi: 10.1371/journal.pone.0286374 (PMC10228813; doi:10.1371/journal.pone.0286374)
Supplement: Attachment — Submitted filename: COMMENTS ON MANUSCRIPT TITLED WTP FOR HI IN ZIMBABWE.docx [file pone.0286374.s004.docx]

**COMMENTS ON MANUSCRIPT TITLED - POTENTIAL DEMAND FOR HEALTH INSURANCE IN ZIMBABWE: EVIDENCE FROM INFORMAL SECTOR CLUSTERS IN HARARE**

**GENERAL COMMENTS**

- Health decisions are mainly decisions made at the household level especially for poor and informal sector households. So why did you use individuals as against households as your economic agent. Please explain briefly even in two sentences your choice of economic agents.
- You need to define every acronym you used
- Please go over your methodology especially on WTP and Marginal WTP for the NHI.
- In your methodology, you did not explain any model and how you came to the conclusion of using the regression methods you chose.
- Generally, you need to explain with reasons why your choice of a particular method over the others.
- Also ensure that the values in the results are what’s being explained in your analysis.

**SPECIFIC COMMENTS**

**Abstract** – In your methods, you failed to show the exact category of Informal Sectors you included in the study. It will be good to know those categories.

**Study Design** – As stated above, there is need to know the informal sectors we’re talking of here. So please list/name them for better understanding.

**Eliciting Willingness to Join and Pay**

- The word contingent in first sentence is the wrong word in that sentence. Rephrase the sentence with the right word to show exactly what you want to explain.
- The DBDC with follow up is not properly explained. So, add few sentences to make it clearer for the reader. Note, it is the basis for the estimation techniques and results used in this work.
- Line 6 in paragraph 3 should be rephrased to read thus: If the respondent indicated WTP, a higher bid was offered as the starting premium.
- In your methodology, you did not state reasons why you choose the DBDC with follow up above the other CV methods.

**Willingness to Join and Pay**

What is the rationale for using both regression methods – Logit and Probit Models. You need to explain why the decision for using both models. I’ll prefer you use one over the other but with a reason.

**Results**

It is confusing having some tables and the main tables that should be shown in the work are not there. Instead, you have directed us the readers to insert S3, S4, S5 etc. to see the tables but others where shown in the text. Be consistent in that and preferably show the main tables and the others send them to an appendix.

**Univariate Analysis**

Those individuals that did not want to join the health insurance scheme, did they give any reason? If yes, it will be nice to know those reasons.

**Willingness to Pay for NHI**

- Respondents who were WTJ were presented with the initial bid randomly drawn from 4 bids where the respondents tend to state their maximum WTP amount. The amount can take the form of any of those 4 bids; hence a Multinomial Logit Model can be appropriate here since it can handle more than two choices.
- Note also that when the DBDC with follow up model is used, we get 4 possible outcomes, with probabilities thus:

Pr. (Yes, Yes)

Pr. (Yes, No)

Pr. (No, Yes)

Pr. (No, No)

You model this and use a loglikelihood function. Thus, an OLS is not the appropriate method here also. There are other estimation methods to use, check the literature properly.
