# Supplementary material for: Potential demand for National Health Insurance in Zimbabwe: Evidence from selected urban informal sector clusters in Harare
Source: PLoS One. 2023 May 30;18(5):e0286374. doi: 10.1371/journal.pone.0286374 (PMC10228813; doi:10.1371/journal.pone.0286374)
Supplement: Attachment — Submitted filename: Response to Reviewers.docx [file pone.0286374.s005.docx]

**COMMENTS ON MANUSCRIPT TITLED - POTENTIAL DEMAND FOR HEALTH INSURANCE IN ZIMBABWE: EVIDENCE FROM INFORMAL SECTOR CLUSTERS IN HARARE**

**GENERAL COMMENTS**

- Health decisions are mainly decisions made at the household level especially for poor and informal sector households. So why did you use individuals as against households as your economic agent. Please explain briefly even in two sentences your choice of economic agents.

Response

It’s true that health decisions are made at the household level, and normally the household head responds to the questionnaire on behalf of the family. However, in the current study it was not possible to collect data exclusively from household heads since data collection happened at the workplaces rather than at homes. It was not possible to apply probability sampling at homes because such sampling frame was unavailable; the Harare City Council’s Human Capital Department could only provide statistics on the number of stalls by informal sector cluster and this information enabled the use of probability sampling to select respondents at their workplace. The use of probability sampling is in line with the best practice for stated preference studies. Moreover, no children under 18 years old nor the elderly (over 64 years) responded to the questionnaire. Nevertheless, failure to interview strictly household heads only has been documented as one of the study limitations (see the revised manuscript).

- You need to define every acronym you used

Response

All acronyms have now been defined – see the revised manuscript.

- Please go over your methodology especially on WTP and Marginal WTP for the NHI.

Response

Models for WTJ and WTP have been specified and described (see the revised manuscript)

- In your methodology, you did not explain any model and how you came to the conclusion of using the regression methods you chose.

Response

The logit model for Willingness to Join and Pay the first bid, and the interval data model for maximum WTP have now been presented and described in the revised manuscript.

- Generally, you need to explain with reasons why your choice of a particular method over the others.

Response

The use of certain regression methods over others has now been explained (see the revised manuscript).

- Also ensure that the values in the results are what’s being explained in your analysis.

Response

All interpretations have been crosschecked to ensure that they are in line with the values reported in the results.

**SPECIFIC COMMENTS**

**Abstract** – In your methods, you failed to show the exact category of Informal Sectors you included in the study. It will be good to know those categories.

Response

The names of the informal sector clusters studied have also been included in the abstract (see the revised manuscript). Also, the word “urban” has been included in the manuscript title and whenever the informal sector clusters are mentioned so that readers can easily recognize that Harare is an urban area.

**Study Design** – As stated above, there is need to know the informal sectors we’re talking of here. So please list/name them for better understanding.

Response

The names of the informal sector clusters had already been stated under the Study Design section of the original manuscript (see the original and revised manuscripts).

**Eliciting Willingness to Join and Pay**

- The word contingent in first sentence is the wrong word in that sentence. Rephrase the sentence with the right word to show exactly what you want to explain.

Response

The word “contingent” has been replaced to add clarity to the meaning of the statement (see the revised manuscript).

- The DBDC with follow up is not properly explained. So, add few sentences to make it clearer for the reader. Note, it is the basis for the estimation techniques and results used in this work.

Response

Some sentences have been added to make the DBDC with follow-up method clearer to the reader (see the revised manuscript).

- Line 6 in paragraph 3 should be rephrased to read thus: If the respondent indicated WTP, a higher bid was offered as the starting premium.

Response

A higher bid was not offered as the “starting premium”; instead, the higher bid was offered as a “follow up to accepting the initial bid” – this is highlighted in section D of the questionnaire. In the revised manuscript, the researcher has provided further details to clarify this issue.

- In your methodology, you did not state reasons why you choose the DBDC with follow up above the other CV methods.

Response

The relative merits of the DBDC with follow-up method in comparison to other elicitation formats have now been highlighted (see the revised manuscript).

**Willingness to Join and Pay**

What is the rationale for using both regression methods – Logit and Probit Models. You need to explain why the decision for using both models. I’ll prefer you use one over the other but with a reason.

Response

There was no big reason for estimating both the logit and probit models. In the revised manuscript, only the logit model was estimated as it allowed the researcher to report and interpret the odds ratios as well; the goodness of fit of the logit and probit models was generally the same.

**Results**

It is confusing having some tables and the main tables that should be shown in the work are not there. Instead, you have directed us the readers to insert S3, S4, S5 etc. to see the tables but others where shown in the text. Be consistent in that and preferably show the main tables and the others send them to an appendix.

Response

The researcher placed some tables as “inserts” to avoid making the manuscript relatively long. In the revised manuscript, most tables are now shown in the text – only those for the odds ratios remain in the appendix.

**Univariate Analysis**

Those individuals that did not want to join the health insurance scheme, did they give any reason? If yes, it will be nice to know those reasons.

Response

The researcher did not make follow-ups to get the reasons for not willing to join.

**Willingness to Pay for NHI**

- Respondents who were WTJ were presented with the initial bid randomly drawn from 4 bids where the respondents tend to state their maximum WTP amount. The amount can take the form of any of those 4 bids; hence a Multinomial Logit Model can be appropriate here since it can handle more than two choices.

Response

The multinomial logit was not applicable because the respondent was not given the opportunity to choose among the 4 initial bids; instead, only one initial bid was offered to the respondent depending on the questionnaire that was randomly drawn by the researcher. Thus, the first WTP response in the “one and only one” questionnaire presented to the respondent was binary (*yes* or *no* answer). In the logit model for WTP, the initial bid price was included as one of the control variables. As highlighted in the original and the updated manuscripts, the researcher prepared 4 sets of questionnaires which only differed on the bids offered (section D) and this was done to minimize anchoring bias. For further clarification, the 4 versions of the questionnaire (which only differ on section D) are embedded as a single document here and the same has been attached as supporting information:

- Note also that when the DBDC with follow up model is used, we get 4 possible outcomes, with probabilities thus:

Pr. (Yes, Yes)

Pr. (Yes, No)

Pr. (No, Yes)

Pr. (No, No)

You model this and use a loglikelihood function. Thus, an OLS is not the appropriate method here also. There are other estimation methods to use, check the literature properly.

Response

It’s true that with the DBDC elicitation there are 4 possible outcomes, and estimation should be done via maximum likelihood since the regressand will be bounded between two points. However, it is argued that if respondents also state their maximum Willingness to Pay (WTP), the regressand will be a continuous variable with a single point estimate such that the OLS technique can be applied (https://doi.org/10.3390/su132111711). Studies that elicited WTP using the DBDC method and estimated WTP via OLS include https://doi.org/10.3390/su132111711; https://doi.org/10.1186/s12939-020-01343-9; and https://doi.org/10.3389/fpubh.2021.654362.

In the revised manuscript, a maximum likelihood approach (interval regression) was applied for the DBDC WTP model, and the results validated those of the OLS technique adopted earlier.

Thank you
