# Supplementary material for: Potential demand for National Health Insurance in Zimbabwe: Evidence from selected urban informal sector clusters in Harare
Source: PLoS One. 2023 May 30;18(5):e0286374. doi: 10.1371/journal.pone.0286374 (PMC10228813; doi:10.1371/journal.pone.0286374)
Supplement: Attachment — Submitted filename: Response to Reviewers.docx [file pone.0286374.s006.docx]

PONE-D-22-35610R1

Potential demand for National Health Insurance in Zimbabwe: evidence from selected urban informal sector clusters in Harare

PLOS ONE

Dear Dr. Chipunza,

Thank you for submitting your manuscript to PLOS ONE. After careful consideration, we feel that it has merit but does not fully meet PLOS ONE’s publication criteria as it currently stands. Therefore, we invite you to submit a revised version of the manuscript that addresses the points raised during the review process.

Please address the following minor comments before it is forwarded for publication.

-Could you include the point of data collection (household or workplaces or both??) in the abstract and under methods. In an informal sector, a workplace can also be a place of residence.

Response

The point of data collection (that is, a workplace) has been specified in the abstract and under methods. (See the revised manuscript).

- also, let the reader know how the sampling was done (in the abstract). You indicate systematic sampling, but you don't tell how systematic it was and was it uniform across all clusters? Also, how were the clusters selected? purposively or randomly? and what was the sampling frame and how did you allocate the sample size? if it was purposive, please give a reason.

Response

The use of purposive sampling in the first stage, the nature of the systematic sampling applied thereafter, and the proportional allocation of the sample based on cluster size have been described in both the abstract and the methods section. Justification for using purposive sampling in the first stage has also been provided in the methods section. (See the revised manuscript).

- I am thinking it would be nice to include some socio-economic (income, occupation, education) include age of your participants in the abstract.

Response

Information on the occupation, education, income, and age of respondents has been included in the abstract (see the revised manuscript).

- Please make all the variables in the table clear. for instance, Hhsize (I am supposing household size).

Response

Full words or appropriate phrases (rather than abbreviations) have been used to describe variables in all the tables. (See the revised manuscript).

- It would have been nice to generate the marginal probability graphs for some of the continuous variables (household size, age, distance) and with other variables with at least three categories (income, education level).

Response

Graphs for some categorical variables (income, education level, employment status), and the continuous variables (household size, age, distance) have been generated (see Figs 1A-1C and Figs 2A-2C of the revised manuscript).

-Conclusion (both abstract and main text) and recommendations should be based on your finding. Kindly tailor this to the study findings

Response

Clarity has been added to ensure that the conclusions and recommendations relate to the findings from informal sector clusters that were studied.

-under background section, I missed statistics on OOP in Zambabwe and the overall health financing system in the country.

Response
Further information on the existing healthcare financing model in Zimbabwe has been provided in the introduction section of the revised manuscript.

Thank you
